# Random measurement error: Why worry? An example of cardiovascular risk factors

**DOI:** 10.1371/journal.pone.0192298

**Published:** 2018-02-09

**Authors:** Timo B. Brakenhoff, Maarten van Smeden, Frank L. J. Visseren, Rolf H. H. Groenwold

**Affiliations:** 1 Julius Center for Health Sciences and Primary Care, University Medical Center Utrecht, Utrecht, the Netherlands; 2 Department of Vascular Medicine, University Medical Center Utrecht, Utrecht, the Netherlands; State University of Rio de Janeiro, BRAZIL

## Abstract

With the increased use of data not originally recorded for research, such as routine care data (or ‘big data’), measurement error is bound to become an increasingly relevant problem in medical research. A common view among medical researchers on the influence of random measurement error (i.e. classical measurement error) is that its presence leads to some degree of systematic underestimation of studied exposure-outcome relations (i.e. attenuation of the effect estimate). For the common situation where the analysis involves at least one exposure and one confounder, we demonstrate that the direction of effect of random measurement error on the estimated exposure-outcome relations can be difficult to anticipate. Using three example studies on cardiovascular risk factors, we illustrate that random measurement error in the exposure and/or confounder can lead to underestimation as well as overestimation of exposure-outcome relations. We therefore advise medical researchers to refrain from making claims about the direction of effect of measurement error in their manuscripts, unless the appropriate inferential tools are used to study or alleviate the impact of measurement error from the analysis.

## Introduction

Measurement error is one of the key challenges to making valid inferences in clinical research [1]. Errors in measurements can arise due to inaccuracy or imprecision of measurement instruments, single measurements of variable longitudinal processes, or non-adherence to measurement protocols. With the increased use of data not originally recorded for research, such as routine care data (or ‘big data’), measurement error is bound to become increasingly relevant in this field [2]. Despite multiple cautionary notes against it [3–13], a common view on the influence of measurement error is that it leads to systematic underestimation of the studied exposure-outcome relations (i.e. attenuation of effect or regression dilution bias) [14]. Using three illustrative example studies on cardiovascular risk factors, we demonstrate that the direction of effect of random measurement error on the estimated exposure-outcome relations can be difficult to anticipate.

### Measurement error in clinical research

Consider the measurement of blood pressure (BP). According to European guidelines for the management of arterial hypertension [15], accurate BP measurement in the clinic using auscultatory or oscillometric semiautomatic sphygmomanometers requires a patient to remain seated for 3 to 5 minutes before taking at least two measurements spaced 1–2 minutes apart. While these stringent measurements procedures of BP are feasible in some highly controlled research settings, it is not difficult to imagine how time constraints and other factors in routine care may cause non-adherence to the BP measurement protocol [16–19]. This non-adherence can lead to the presence of measurement error in routinely recorded BP measurements. Other obvious sources of measurement error in these measurements are the known imperfect accuracy of sphygmomanometers [20] and the white-coat effect [21].

Now consider a study of BP as a possible risk factor for developing cardiovascular disease. Data analysis based on routine care BP data can evidently suffer when routine BP measurements are systemically lower or higher than actual BP, or when the measurement error depends on patient characteristics (e.g. more or less measurement error in older individuals). However, when measurement error in BP is a completely random process—known as *classical error* [22]—the potential impact of measurement error becomes less apparent. A common view on the influence of such random error in risk factors (i.e. exposures) is that its presence leads to attenuation of the exposure-outcome relation. Intuitively, in the context of BP, when the recorded BP measurements are more variable (contains more ‘noise’) due to measurement error, the BP-cardiovascular disease relation becomes obscured (i.e. attenuated), as compared to what would have been observed with ideal measurement of BP in the same individuals (the theoretical gold standard, without ‘noise’).

In general, under this *attenuation of the effect* “assumption”, the estimated effects of exposure-outcome relations in the presence of measurement error are considered conservative estimates (where conservativeness increases as the amount of error increases) of the counterfactual situation where measurement error would be absent, paradoxically, leading to the notion that estimates found in data with more measurement error are more credible than data without measurement error (“that which does not kill statistical significance makes it stronger”,[7]). Many authors[3–13] before us have warned that attenuation is by no means guaranteed to occur (even when the measurement error in the exposure classifies as simple classical error) and that the magnitude and direction of bias due to measurement error on the exposure-outcome effect estimate is typically difficult to estimate without applying specialized statistical methods. However, in a systematic review[23], of recent publications in top-ranked general medicine and epidemiology journals (*N = 565*) we found that *attenuation of effect* remains a prevailing notion among medical writers, which almost always remains unsubstantiated by their statistical analyses.

To re-emphasize the unpredictable impact of random error in a medical context, we show three illustrative examples of estimating risk of cardiovascular disease using a conventional Cox proportional hazards model. Our result also easily extends to other diseases and other (non-linear) statistical models.

## Materials and methods: Risk factors for cardiovascular events

Data of 7,395 patients with manifest vascular disease from the Second Manifestations of ARTerial disease (SMART) cohort [24] aged 35 years or older and with complete data on the variables relevant to our study were included in our analyses (Table 1). In short, the SMART study is a prospective single-center cohort study which started recruiting patients in 1996. The primary aim was studying the prevalence and incidence of additional cardiovascular disease in patients who experienced a manifestation of arterial disease or who are otherwise at a high risk to develop symptomatic arterial disease [24].

**Table 1:** Baseline characteristics of the example dataset of patients with manifest vascular disease.

Baseline characteristic	N = 7395
**Age in years (mean (sd))**	60.5 (9.7)
**Male (%)**	5474 (74)
**SBP in mmHg (mean (sd))**	140 (21)
**DBP in mmHg (mean (sd))**	81 (11)
**CIMT in mm (mean (sd))**	0.92 (0.27)
**ABI (mean (sd))**	1.09 (0.19)
**Follow up in days (median [IQR])**	2510 [1293–3827]
Cardiovascular events* **during follow up (%)**	1309 (18)

SBP = systolic blood pressure; DBP = diastolic blood pressure; CIMT = carotid intima media thickness; ABI = ankle-brachial index at rest; IQR = interquartile range.

*Defined as the composite of myocardial infarction, stroke, and cardiovascular death (whichever came first) developed over a minimum of three years of follow up time.

For our studies, we focused on two established exposure-outcome relations: (1) systolic blood pressure (SBP) and cardiovascular events and (2) carotid intima media thickness (CIMT) and cardiovascular events. SBP (in mmHg) and CIMT (in mm) were measured at cohort enrollment. Cardiovascular events were defined as the composite of myocardial infarction, stroke, and cardiovascular death (whichever came first) developed within a minimum of three years after cohort enrolment. The following confounders of both these relations were considered and measured at cohort entry: diastolic blood pressure (DBP; in mmHg); ankle-brachial index at rest (ABI); age; and sex. A total of three multivariable models were considered with SBP (in models 1 and 2) and CIMT (model 3) as the exposure variable. DBP, ABI, and SBP were included as confounders in models 1–3, respectively. Age and sex were included as confounders in all three models. A Cox proportional hazards survival model was used to estimate the crude and confounder adjusted hazard ratios (HR) of the exposure and main confounder in each model. The proportional hazards assumption was assessed through visual inspection of the Martingale residuals (no evidence of deviations from the assumption were found).

### Generating measurement error

The original recordings of the variables (SBP, CIMT, ABI, DBP, age and sex) in the SMART cohort were assumed to be without error. To illustrate the impact of random measurement error in exposure and/or confounders, three separate scenarios were evaluated. Specifically, measurement error was artificially added to the original exposure (either SBP or CIMT) and/or one of the confounders (specifically ABI, DBP or SBP, in models 1–3, respectively) by adding measurement errors that were randomly drawn from a normal distribution with mean zero. The measurement error thus increased a variable′s variance, but did not influence its mean. This type of measurement error satisfies the criteria for classical error [22]. No measurement error was added to the confounders age and sex. To reduce the impact of chance phenomena, each scenario was repeated 1,000 times and results were averaged on the log hazard ratio scale. The obtained average HRs were then compared to *reference* HRs calculated in the original data (i.e. without measurement error). Simulations and analyses were performed in the statistical software program *R* (v. 3.12) [25].

## Results

Table 2 shows the unadjusted and confounding adjusted HRs for a cardiovascular event of the exposures SBP and CIMT as well as the main confounders (DBP, ABI, and SBP) when analyzing the original data. The HR of SBP per 10 mmHg (HR = 1.11, 95% CI: 1.09 to 1.14) slightly decreased to HR 1.10 (95% CI: 1.07 to 1.14) after adjustment for age, sex, and DBP (model 1) and to HR 1.03 (95% CI: 1.00 to 1.06), after adjustment for age, sex, and ABI (model 2). Similarly, the HR of CIMT decreased from 2.82 (95% CI: 2.48 to 3.20) to 2.10 (95% CI: 1.79 to 2.47) when adjusting for age, sex, and SBP (model 3). The main confounders in model 1 (DBP) and 2 (ABI) had negative relationships with the outcome. To further investigate the confounding structure of the main confounders, the Pearson correlation coefficient between the main confounder and exposure in each model was calculated (in the absence of simulated measurement error). The correlation between SBP and DBP in model 1 was 0.65; the correlation between SBP and ABI in model 2 was -0.17; and the correlation between CIMT and SBP in model 3 was 0.25.

**Table 2:** Crude and adjusted hazard ratios for the relation of the exposures (SBP and CIMT) and main confounders (DBP, ABI, and SBP) with the outcome (cardiovascular events).

Model	Variable	Crude HR (95% CI)	Adjusted HR* (95% CI)	
**1**	Exposure: SBP per 10 mmHg	1.11 (1.09, 1.14)		1.10 (1.07, 1.14)
	Confounder: DBP per 10 mmHg	0.99 (0.94, 1.04)		0.88 (0.83, 0.94)
**2**	Exposure: SBP per 10 mmHg	1.11 (1.09, 1.14)		1.03 (1.00, 1.06)
	Confounder: ABI	0.20 (0.16, 0.26)		0.22 (0.18, 0.29)
**3**	Exposure: CIMT per mm	2.82 (2.48, 3.20)		2.10 (1.79, 2.47)
	Confounder: SBP per 10 mmHg	1.11 (1.09, 1.14)		1.04 (1.01, 1.06)

HR = hazard ratio; SBP = systolic blood pressure; CIMT = carotid intima media thickness; DBP = diastolic blood pressure; ABI = ankle-brachial index at rest.

*Besides the exposure and main confounder shown in the table, each model was further adjusted for the variables age and sex.

**Fig 1:**
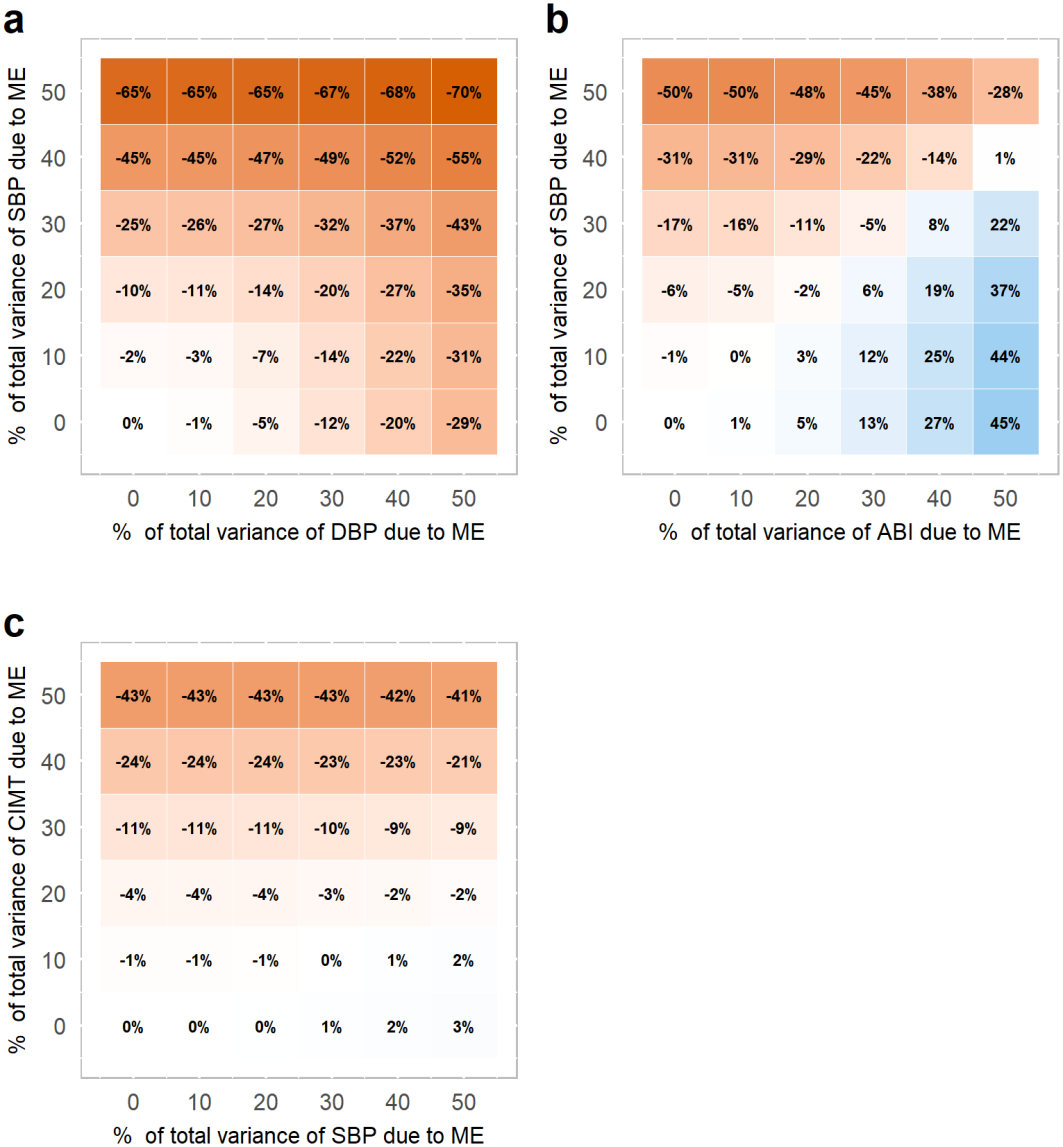
Relative bias of the exposure-outcome relation when the exposure and confounder contain random measurement error. The relative bias is expressed as a % of the adjusted exposure-outcome relation when there is no ME (reference standard; see Table 2). The amount of added ME is expressed as a percentage of the total variance of the variable. In (a) and (b) ME is added to the exposure, SBP, and to a confounder; DBP in (a) or ABI in (b). In (c) ME is added to the exposure, CIMT, and a confounder, SBP. Age and sex were additionally included as confounders for all multivariable analyses. Red colors indicate and an underestimation of the exposure-outcome relation due to ME, whereas blue colors indicate an overestimation. ME = measurement error; SBP = systolic blood pressure; DBP = diastolic blood pressure; ABI = ankle-brachial index; CIMT = carotid intima media thickness.

Fig 1 illustrates the impact of measurement error in the exposure (vertical axis) and/or confounder (horizontal axis) for each of the three models. The amount of measurement error in an exposure or confounder is expressed as the percentage of the total variance of that variable. For example, when 50% of the total variance of a variable is due to measurement error, this means that the variance of the added measurement error equals the variance of the original variable. Red colors indicate an underestimation of the exposure-outcome relation due to measurement error, whereas blue colors indicate an overestimation.

The exposure-outcome relation of model 1 was attenuated when measurement error was added solely to the exposure variable SBP (Fig 1a). As the amount of measurement error in SBP increased, the exposure-outcome relation was increasingly underestimated. Attenuation of the exposure-outcome relation was also observed when adding measurement error solely to the confounder (DBP). As could be expected, adding measurement error to both SBP and DBP led to the most underestimation of the original exposure-outcome relation.

A different pattern is observed for model 2 (Fig 1b). When solely the exposure SBP was measured with error, the exposure-outcome relation was again attenuated, as was observed for model 1. However, when adding measurement error solely to the confounder ABI, this led to overestimation of the exposure-outcome relation. This is in the opposite direction of that observed in model 1 when adding measurement error to DBP. When measurement error was in both the exposure and the confounder, the combination of effects ranged between severe attenuation and severe exaggeration.

For model 3, there was a negligible effect on the exposure-outcome relation when adding measurement error to the confounder SBP (Fig 1c). As a result, the attenuation of the exposure-outcome relation caused by measurement error in the exposure CIMT was consistent across different levels of measurement error in the confounder SBP.

## Discussion and conclusion

Our illustrative examples re-emphasize that random measurement error in exposure or confounders does not automatically result in an attenuation of the exposure-outcome relation. In fact, it can be difficult to anticipate the direction of effect of random measurement error on the exposure-outcome relations in common settings with at least one exposure and one confounder. That is, depending on the relationship of the confounder with the exposure and the outcome, as well as the type and magnitude of measurement error on the exposure and/or confounder, the exposure-outcome relation may be attenuated, exaggerated or remain unaffected due to the measurement error.

The different effects of classical measurement error on the estimated relations can be explained by the interplay of at least two factors besides the magnitude of measurement error. First, the magnitude and direction of the correlation between variables can alter the direction of the effect of measurement error [8,9,12]. For instance, in our study we found a switch of direction of effect when considering a negatively versus a positively correlated exposure-confounder relation. Another factor is the strength of the relationship between the confounder and the outcome [12]. The impact of measurement error in the confounder on the estimated exposure-outcome relations thus depends on the actual confounding structure.

While it is already challenging to predict the direction and magnitude of bias in the presented illustrative examples, in practice this can become even more complex, as more interrelated variables can be added to the analysis model which to different extents may be suffering from some degree of measurement error. Obviously, more complex measurement error structures than classical error may be considered, such as when dealing with correlated measurement errors [11], interaction terms [13] or differential errors [3–5].

An additional remark can be made about the presented examples. To investigate the effect of measurement error on the studied relations, we considered the original variables to be measured without error. Measurement error was then added artificially in the different scenarios. While measurements of, e.g., SBP, CIMT or ABI are standardized in practice, actual measurements may still not adequately capture the phenomenon of interest. As detailed for BP in the introduction, inaccuracy or imprecision of measurement instruments and non-adherence to measurement protocols are all reasons why routinely collected measurements will differ from those sought for specific research purposes. The examples presented here merely serve illustration purposes.

We believe that authors should be cautious when making statements concerning the possible impact of measurement error on the direction of effect in the studied relation, without supporting evidence. One step beyond hypothesizing the direction and magnitude of the impact of measurement error is to correct for it. A range of techniques is available, such as regression calibration [26,27], simulation extrapolation (SIMEX) [28] and probabilistic sensitivity analyses [1]. We refer to the literature for a description of these and other methods [1,22,29,30].

In conclusion, the commonly held belief that random measurement error leads to a systematic underestimation (‘attenuation’) of exposure-outcome relations and thus yields conservative estimates of exposure effects is a simplification of reality, and simply not true in many situations that may be encountered in observational clinical research. With the increasing use of routinely collected health care data for medical research, renewed attention for the complex impact of measurement error and approaches for dealing with measurement error are vital. In addition to comprehensive textbooks [22,30], more applied literature [1,29,31] is available that can aid researchers to account for measurement error during analysis, when it cannot be prevented during data collection.
